# Pepcan-12 (RVD-hemopressin) is a CB2 receptor positive allosteric modulator constitutively secreted by adrenals and in liver upon tissue damage

**DOI:** 10.1038/s41598-017-09808-8

**Published:** 2017-08-25

**Authors:** Vanessa Petrucci, Andrea Chicca, Sandra Glasmacher, Janos Paloczi, Zongxian Cao, Pal Pacher, Jürg Gertsch

**Affiliations:** 1 Institute of Biochemistry and Molecular Medicine, University of Bern, Bühlstrasse 28, 3012 Bern, Switzerland; 2 Graduate School for Cellular and Biomedical Sciences, University of Bern, Bern, Switzerland; 3 Laboratory of Cardiovascular Physiology and Tissue Injury, National Institutes of Health/NIAAA, Bethesda, MD USA

## Abstract

Pepcan-12 (RVD-hemopressin; RVDPVNFKLLSH) is the major peptide of a family of endogenous peptide endocannabinoids (pepcans) shown to act as negative allosteric modulators (NAM) of cannabinoid CB1 receptors. Noradrenergic neurons have been identified to be a specific site of pepcan production. However, it remains unknown whether pepcans occur in the periphery and interact with peripheral CB2 cannabinoid receptors. Here, it is shown that pepcan-12 acts as a potent (*K*_*i*_ value ~50 nM) *h*CB2 receptor positive allosteric modulator (PAM). It significantly potentiated the effects of CB2 receptor agonists, including the endocannabinoid 2-arachidonoyl glycerol (2-AG), for [35S]GTPγS binding and cAMP inhibition (5–10 fold). In mice, the putative precursor pepcan-23 (SALSDLHAHKLRVDPVNFKLLSH) was identified with pepcan-12 in brain, liver and kidney. Pepcan-12 was increased upon endotoxemia and ischemia reperfusion damage where CB2 receptors play a protective role. The adrenals are a major endocrine site of production/secretion of constitutive pepcan-12, as shown by its marked loss after adrenalectomy. However, upon I/R damage pepcan-12 was strongly increased in the liver (from ~100 pmol/g to ~500 pmol/g) independent of adrenals. The wide occurrence of this endogenous hormone-like CB2 receptor PAM, with unforeseen opposite allosteric effects on cannabinoid receptors, suggests its potential role in peripheral pathophysiological processes.

## Introduction

The two major arachidonic acid-derived endocannabinoids 2-arachidonoyl glycerol (2-AG) and *N*-arachidonolyethanolamide (AEA) are lipids that occur widely in nature^1–3^. They have been extensively studied in mammalian cells and tissues where they act as non-selective CB1 and CB2 cannabinoid receptor agonists at discrete transmembrane orthosteric binding sites^4–6^. Although CB1 receptors are abundantly expressed in the brain, they are also co-expressed in peripheral tissues together with peripheral (type-2) CB2 cannabinoid receptors, which are primarily present in immune cells^7–10^. While CB2 receptor activation is generally associated with tissue protective and inflammation modulating effects^11–14^, chronic activation of CB1 receptors in peripheral tissues may promote hyperlipidemia, diabetes, hepatorenal inflammation and cardiometabolic risk^15–17^. Whether endocannabinoids or non-selective exogenous cannabinoids with comparable agonist potencies and efficacies for both receptors can differentially activate CB1 or CB2 receptors, e.g. when co-expressed in the same cell, remains poorly understood. As outlined here, endogenous allosteric modulators functionally selective for CB1/CB2 receptors could in theory mediate such biased receptor activation. Over the last decade, it has become generally accepted that all G protein-coupled receptors (GPCRs) possess spatially distinct allosteric sites that can be targeted by exogenous substances to modulate the receptors’ functional state^18^. The pharmacological allosteric modulation observed with synthetic ligands likely has its evolutionary origin in the existence of endogenous allosteric modulators as indicated by the increasing number of such molecules identified^19, 20^.

To date, few negative allosteric modulators (NAMs) are known for CB1 receptors^21–23^ and, to our knowledge, no allosteric modulator has hitherto been identified for CB2 receptors. In addition to the reported endogenous CB1 receptor NAM pregnenolone^24^, a family of endogenous peptide endocannabinoids (pepcans), containing the hemopressin amino acid sequence (PVNFKLLSH) from alpha hemoglobin, has been reported to interact with CB1 receptors^25–27^ and RVD-hemopressin (pepcan-12, RVDPVNFKLLSH) was shown to act as CB1 receptor NAM^21, 27^. Originally, the rat hemopressin was reported to be an endogenous CB1 inverse agonist, i.e. antagonist^25^. However, more recent work has shown that this nonapeptide is a hydrolysis artefact generated under acidic conditions and does not occur endogenously^26, 27^.

We have previously shown that mouse/human pepcan-12 acts as a CB1 receptor NAM at the level of cAMP generation and [^35^S]GTPγS binding^27^. These findings have been independently confirmed in experiments measuring synaptic transmission in cultured hippocampal neurons^21^. Like the synthetic CB1 receptor NAMs ORG27569 and PSNCBAM-1 also pepcan-12 attenuated depolarization-induced suppression of excitation (DSE) and did not directly inhibit CB1 receptors^21^. The occurrence of endogenous CB1 receptor NAMs is intriguing as it implies an additional and currently unknown level of control over the endocannabinoid system (ECS). Therefore, the cellular origin and distribution of pepcans, as well as the context of their production/secretion remain to be investigated. Employing the monoclonal antibody (mAb) 1A12 we have recently shown that in the rodent CNS only the noradrenergic neurons (locus coeruleus and A2, A5 and A7 nuclei) display positive pepcan immunofluorescence, clearly labeling the numerous locus coeruleus projecting fibers throughout the brain^28^. In the same study we also found pronounced positive 1A12 immunohistochemical staining in the chromaffin cells of the adrenal medulla. In agreement, the adrenals contained relatively high amounts of pepcan-12 compared to brain as measured by competitive enzyme-linked immunosorbent assays (cELISA) and a LC-MS/MS method^28^. Since chronic CB1 receptor activation is endowed with a negative role in metabolic and inflammatory processes in many peripheral organs^15–17^, we wondered whether peripheral tissues harbor biologically relevant amounts of the CB1 receptor NAM pepcan-12. To that aim, we developed a more suitable LC-MS/MS method for the quantification of pepcans in these tissues. In light of the pronounced non-selective nature of endocannabinoids and the relatively high concentrations of pepcan-12 found in the periphery, in this study we also investigated whether pepcan-12 and other members of the family can modulate the function of the peripheral cannabinoid CB2 (*Cnr2*) receptor. The CB2 receptor shares 43% overall identity with the CB1 (*Cnr*1) receptor, but has little overall homology with other receptors of adjacent phylogenetic clades^29^. Our present data indicate that pepcan-12 is a potent *h*CB2 receptor ligand targeting a binding site different from orthosteric ligands and, in contrast to its effects on *h*CB1 receptors, acts as an efficient *h*CB2 receptor positive allosteric modulator (PAM). Using disease relevant rodent models and adrenalectomized mice, we obtained evidence for the dominant role of adrenals in the endocrine production of pepcan-12, but also found that the liver is able to produce and excrete this peptide in the context of pathophysiological conditions like ischemia reperfusion injury.

## Results

### CB2 receptor binding interaction studies

**Figure 1:**
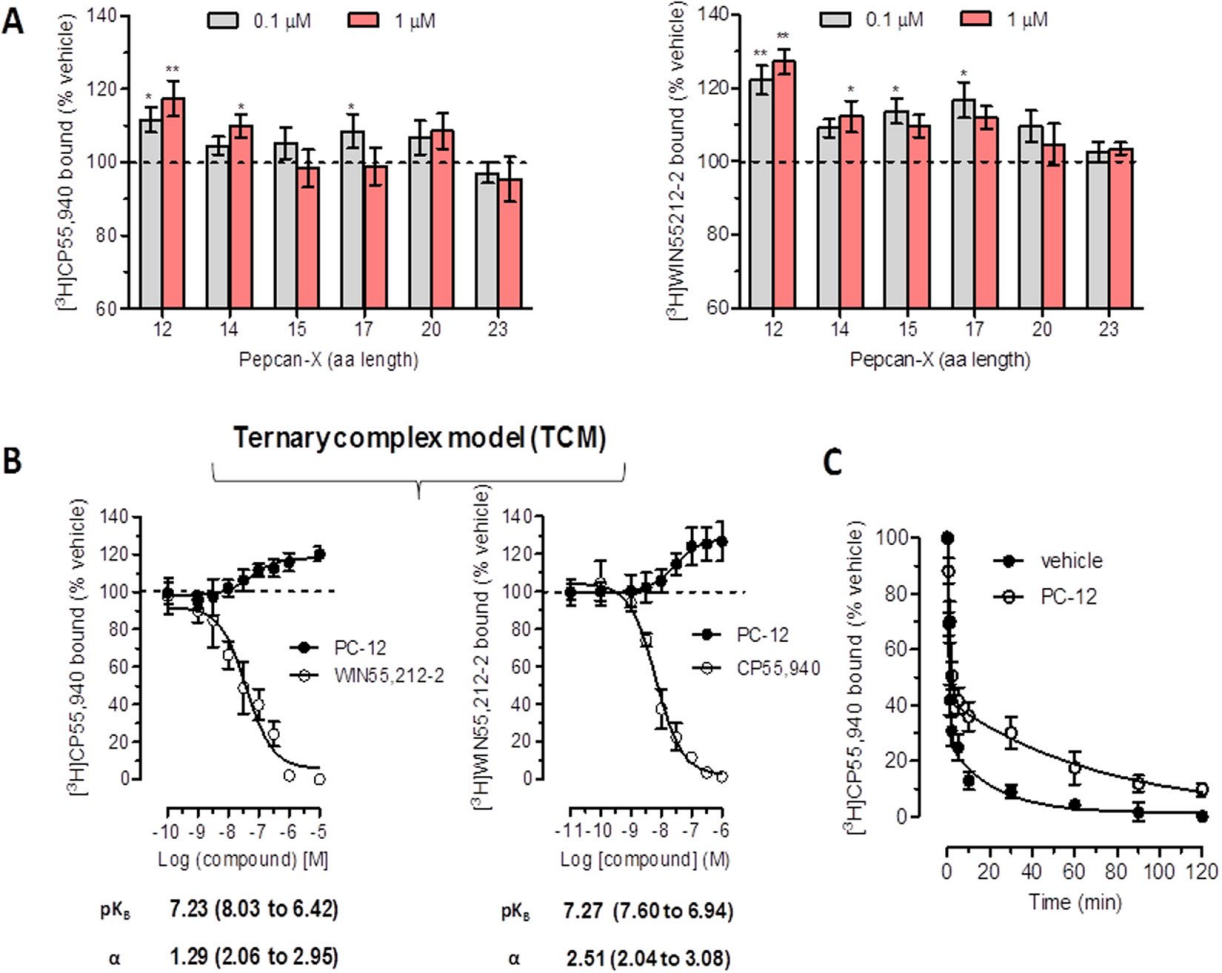
Pepcan binding to *h*CB2 receptors increases binding of orthosteric ligands and positive cooperativity. (**A**) Pepcan-mediated enhancement of [^3^H]CP55940 and [^3^H]WIN55212-2 binding, respectively, to stably transfected *h*CB2 receptor CHO-K1 cell membranes at 100 nM and 1 µM. (**B**) Concentration-dependent potentiation of radioligand binding by pepcan-12 (PC-12) versus mutual competition by the orthosteric ligands showing pK_B_ values and the cooperativity factor α. (**C**) Isotopic dilution in which 1 μM of CP55,940 was added to the CB1 receptor-bound [^3^H]CP55,940 in the presence of 300 nM pepcan-12 or vehicle. Data show mean values ± SEM of at least six independent experiment. *p < 0.05 vs. vehicle (two-tailed student t-test).

The pepcans previously identified in biological tissues (pepcan-12, -14, -15, -17, -20 and -23)^27^ were tested in classical radioactivity-based *h*CB2 receptor binding experiments using [^3^H]CP55,940 and [^3^H]WIN55,212-2 as high-affinity orthosteric CB2 receptor radioligands on stably *h*CB2 receptor transfected CHO cell membranes. Rather unexpectedly, we did not detect radioligand displacement but increased binding in both cases. As shown in Fig. 1A, at the screening concentrations of 100 nM and 1 μM, pepcan-12 was the most potent enhancer of [^3^H]CP55,940 and [^3^H]WIN55,212-2 receptor binding, respectively, inducing an overall significant 20–40% binding increase over vehicle control. Pepcan-14, pepcan-15 and pepcan-17 showed weaker yet still significant effects, while the longest peptides pepcan-20 and pepcan-23 did not exhibit any receptor interaction. In subsequent experiments, pepcan-12 displayed concentration-dependent CB2 receptor binding interactions, as reflected by the concomitant sigmoidal increase of [^3^H]CP55,940 and [^3^H]WIN55,212–2 binding (Fig. 1B). This potent and limited increase (approximately 140%) in CB2 receptor agonist binding suggested a positive allosteric modulation of the orthosteric ligands by pepcan-12 as opposed to the mutual full competition measured between CP55,940 and WIN55,212-2 (Fig. 1B). Therefore, the concentration binding curves obtained with pepcan-12 were analyzed with the ternary complex model that typically describes allosteric modulation^30^. The results provided a cooperativity factor (α) of 1.294 with a K_b_ of 58 nM for [^3^H]CP55,940 and α = 2.513 with a K_b_ of 53 nM for [^3^H]WIN55,212-2. Statistical analyses confirmed that α values were significantly higher than the unit (1), thus confirming the positive cooperation between pepcan-12 and both orthosteric ligands. In order to better characterize the allosteric mechanism postulated, we measured the dissociation kinetics of [^3^H]CP55,940 from CB2 receptors in the presence of pepcan-12. The experiments were performed using an isotopic dilution approach in which 1 μM of CP55,940 was added to the CB2 receptor-bound [^3^H]CP55,940 in the presence of 300 nM pepcan-12 or vehicle. As shown in Fig. 1C, CP55,940 induced a significantly slower dissociation of the bound [^3^H]CP55,940 when incubated in the presence of pepcan-12 as compared to vehicle. Corresponding data analyses provided higher values of both fast *k*_1_ and slow *k*_2_ dissociation rate constants (Table 1).

**Table 1:** Dissociation rate constants at 1 μM of CP55,940 added to CB1 receptor-bound [^3^H]CP55,940 (0.5 nM) in presence of 300 nM pepcan-12 or vehicle.

	*K*_*1*_ (slow)	Half-life (slow)	*K*_2_ (fast)	Half-life (fast)
	x10^−2^ min^−1^	min	min^−1^	min
Vehicle	4.65 ± 1.11	14.91 (10.1 to 28.5)	1.33 ± 0.11	0.521 (0.450 to 0.621)
Pepcan-12	1.39 ± 0.49	49.90 (29.3 to 169.3)	0.82 ± 0.08	0.844 (0.703 to 1.057)

**Figure 2:**
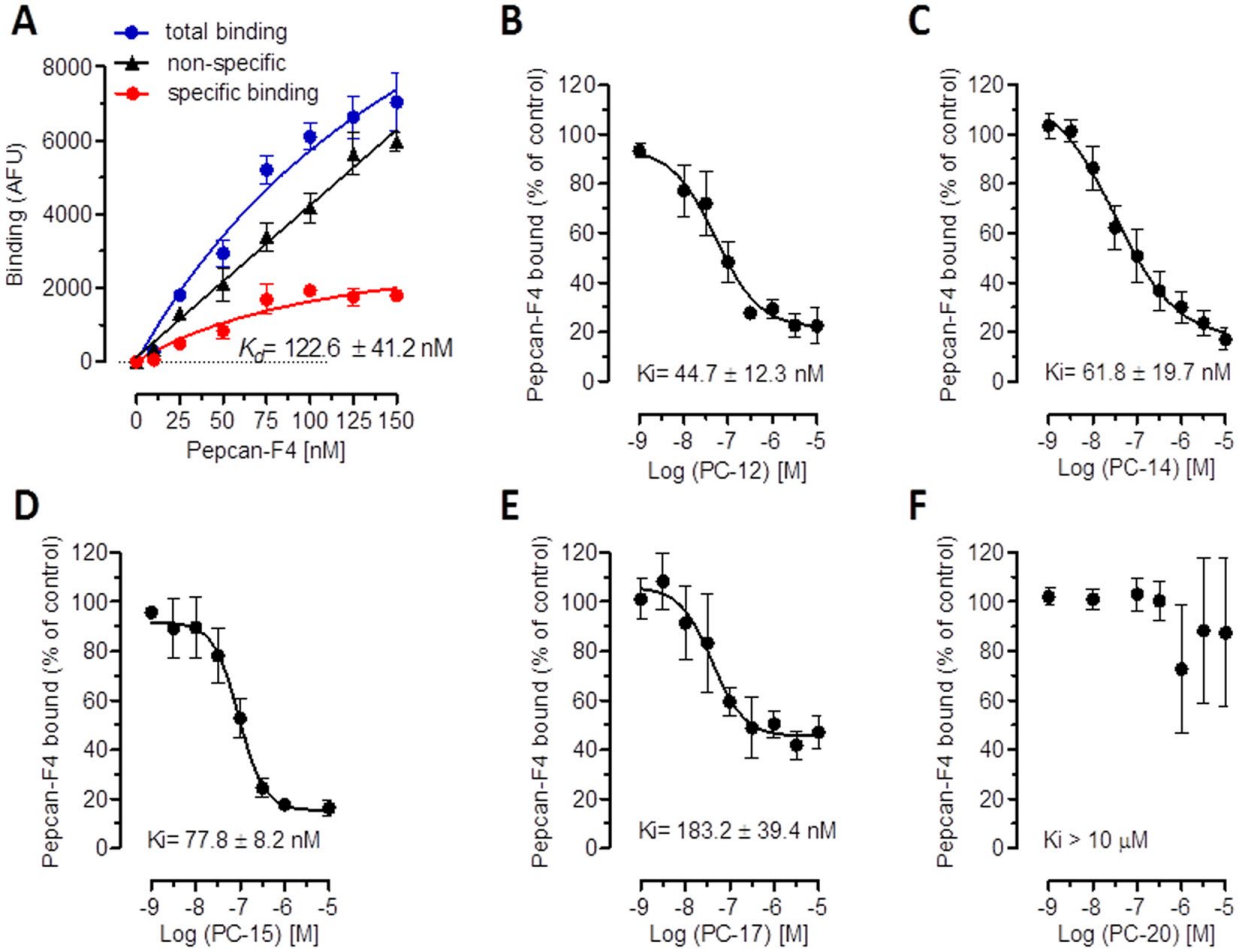
Displacement of fluorescent pepcan-F4 by endogenous pepcans. (**A**) Specific binding of pepcan-F4 to *h*CB2 expressing CHO-K1 cells measured by fluorescence, showing nonspecific binding in CHO-K1 WT cells. (**B**–**F**) Differential dose-dependent displacement of pepcan-F4 by pepcan-12, -14, -15, -17 and -20. Data show mean values of at least three independent experiments each performed in triplicates.

This reduction of [^3^H]CP55,940 dissociation from CB2 receptors was in line with the increased binding of the radioligands by pepcan-12 in the equilibrium binding experiments (Table 1). Both results indicated that pepcan-12 binds to the CB2 receptor at an allosteric binding site. With the intention to further characterize pepcan binding to CB2 receptors, we exploited a C-terminal fluorescence-labeled pepcan-12 conjugate (pepcan-F4), which was previously used to measure pepcan CB1 receptor binding affinities^27^. The apparent *K*_*d*_ value (122 ± 41 nM) for pepcan-F4 for *h*CB2 receptors was calculated from a normalized (specific) saturation curve (Fig. 2A). As shown in Fig. 2B–F, pepcans differentially displaced pepcan-F4, matching the distinct biding interactions measured with the radioligands. Consequently, pepcan-12, −14, 15, and −17 showed nanomolar CB2 receptor binding affinities comparable to the previous experiments, pepcan-12 exhibiting the lowest *K*_i_ value of 44 ± 12 nM. The longest peptide tested (pepcan-20) showed no significant displacement of Pepcan-F4 up to 10 μM (Fig. 2F) in agreement with the lack of CB2 receptor radioligand binding modulation by the N-terminally extended peptide species.

### Pepcan-12 potentiates CP55,940 and 2-AG induced *h*CB2 receptor signaling

Given the allosteric CB2 receptor binding interaction observed with pepcan-12 (Figs 1 and 2), we studied its effects on key signaling events triggered by the CB2 receptor agonists CP55,940 and 2-AG. CP55,940 and the major endocannabinoid 2-AG were selected because both ligands have been shown to act as full agonists at CB2 receptors but display differential signaling bias^31, 32^.

**Figure 3:**
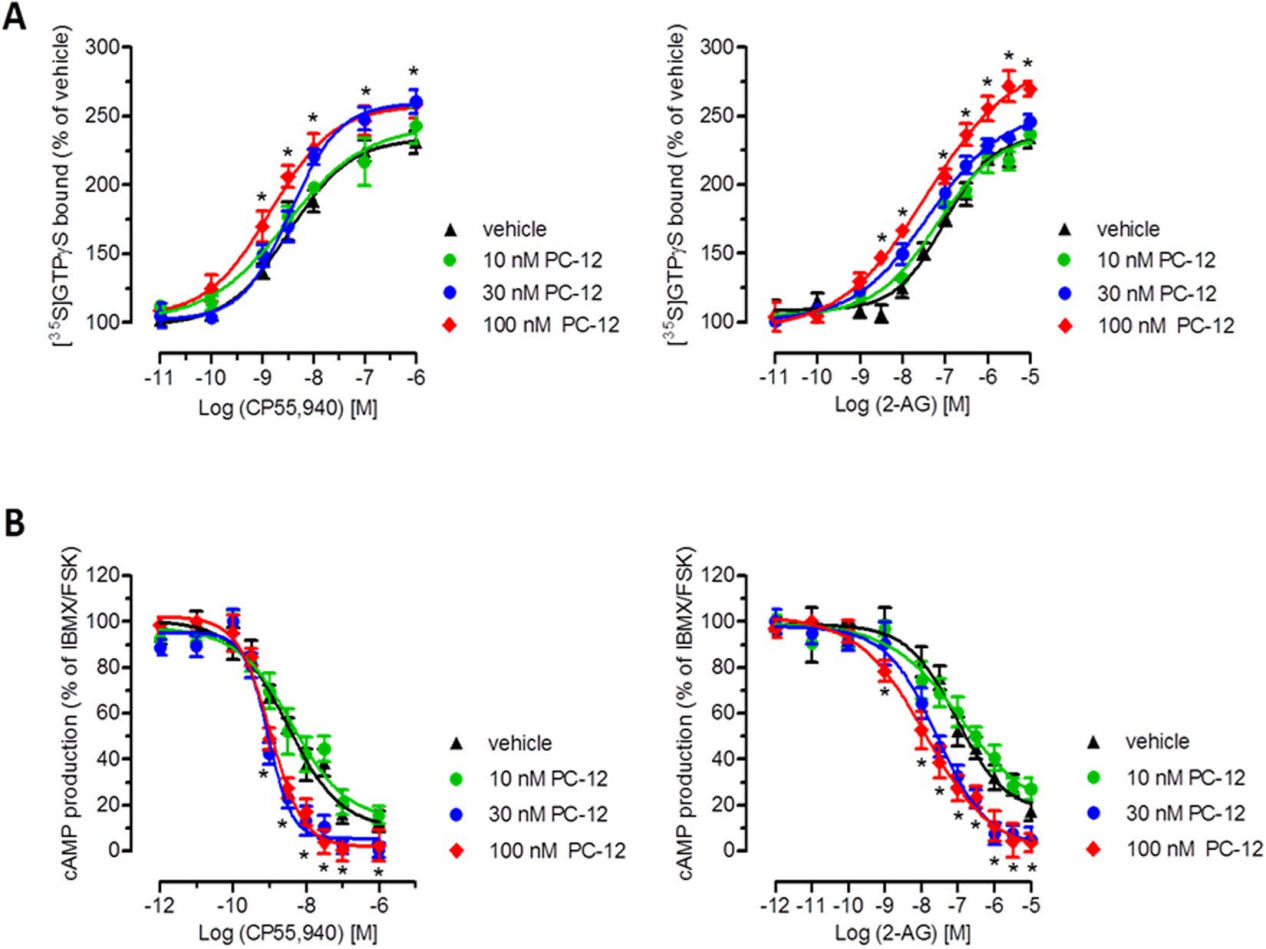
Pepcan-12 potentiates the signaling of CP55,940 and 2-AG. (**A**) Concentration-dependent increase of CB2 receptor agonist [^35^S]GTPγS binding by pepcan-12 in CHO-K1-*h*CB2 cell membranes. (**B**) Concentration-dependent potentiation of cAMP inhibition of CB2 receptor agonists by pepcan-12 in CHO-K1-*h*CB2 cells Data show mean values ± SEM of at least six independent experiments. *p < 0.05 vs. vehicle (two-tailed student t-test).

The effects of pepcan-12 on the modulation of CB2 receptor-mediated signaling was studied by measuring [^35^S]GTPγS receptor binding, cAMP production and β-arrestin2 recruitment. In absence of the orthosteric ligands, pepcan-12 did not trigger or inhibit any of the CB2 receptor-mediated signaling pathways measured (Suppl. Fig. S1A). However, when pepcan-12 was incubated in presence of CP55,940 or 2-AG, it induced a significant potentiation of cAMP inhibition and [^35^S]GTPγS binding (Fig. 3A,B). Pepcan-12 was tested at 10 nM, 30 nM and 100 nM. In line with the estimated *K*_*d*_ value, the concentration of 30 nM induced significant but submaximal effects, while 100 nM elicited maximal effects in both assays.

At 100 nM, pepcan-12 increased CP55,940 and 2-AG affinity for [^35^S]GTPγS binding by approximately 3-fold and led to a higher maximal binding (Fig. 3A, Table 2). In the cAMP assay, the effect of 100 nM pepcan-12 increased the CP55,940-mediated effect by significantly reducing the IC_50_ value from 3.1 nM to 1.0 nM. Intriguingly, pepcan-12 exerted an even stronger potentiation of 2-AG-mediated cAMP inhibition, reducing the EC_50_ value approximately by 8-fold, from 92.1 nM to 12.4 nM (Fig. 3B, Table 2). The other members of the pepcan family did not significantly affect the agonist-induced cAMP inhibition, in line with their lower binding affinities (Suppl. Fig. S2). Unlike for cAMP and [^35^S]GTPγS assays, pepcan-12 (100 and 300 nM) did not influence CP55, 940-induced β-arrestin2 recruitment at the time-point measured (Suppl. Fig. S3). Collectively, these data suggest that pepcan-12 might act as a signaling-specific positive allosteric modulator for CB2 receptor-mediated G protein recruitment and cAMP inhibition (G_αi_), possibly without affecting β-arrestin2 recruitment and receptor internalization.

**Table 2:** Summary of pepcan-12 modulation of CP55,940- and 2-AG-induced G protein activation and cAMP formation.

	[^35^S]GTPγS assay				cAMP assay	
	CP55,940		2-AG		CP55,940	2-AG
Pepcan-12 (nM)	EC_50_ (nM)	Max. Effect (% of crtl)	EC_50_ (nM)	Max. Effect (% of crtl)	EC_50_ (nM)	EC_50_ (nM)
0	3.5 (2.8–6.2)	234 (222–246)	95.8 (66–145)	235 (220–251)	3.1 (1.8–6.7)	92.1 (40–102)
10	3.4 (1.2–8.1)	242 (214–271)	64.9 (32–130)	238 (221–251)	4.1 (1.6–9.1)	102.1 (55–225)
30	2.9 (2.2–5.5)	259 (247–274)	40.9 (20–61)	254 (237–272)	0.9 (0.6–1.3)	25.1 (12–38)
100	1.4 (0.6–2.7)	260 (249–276)	35.4 (16–54)	291 (265–318)	1.0 (0.6–1.5)	12.4 (4–34)

*n* = 6–12; data show means values and 95% CI. *p < 0.05; **p < 0.001.

### Presence of pepcan-12 and pepcan-23 in peripheral tissues and induction of pepcan-12 upon endotoxin challenge

**Figure 4:**
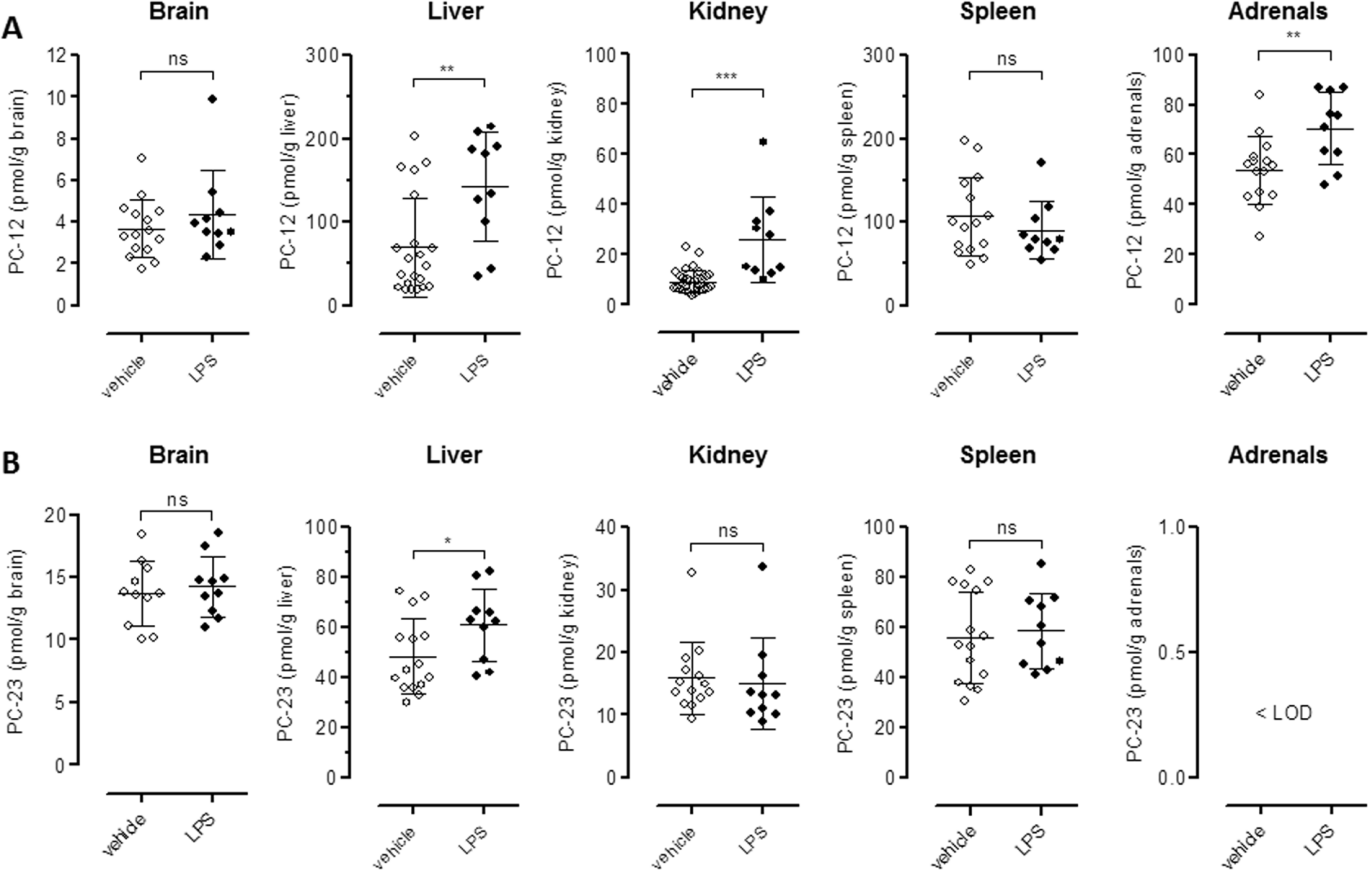
LC-MS/MS quantification of pepcan-12 and pepcan-23 in peripheral tissues upon LPS challenge (6 h) in Swiss albino female mice. Quantification of (**A**) PC-12 and (**B**) PC-23 in tissues of wild type Swiss albino female mice in normal, saline (empty circles) and endotoxemic, 5 mg/kg LPS E.coli 055:B5 serotype (closed circles) conditions after 2 h. Data show means and SD. Groups were compared using two-tailed *t*-Student’s; *p < 0.05; **p < 0.001; ***p < 0.0001; ns = not significant. < LOD, below limit of detection.

For the quantification of pepcans, we initially applied a cELISA method with the IgG2A mAb 1A12 against the C-terminal part (FKLLSH) of pepcans^27^. Using this method we measured an increase of pepcans after 2 h from lipopolysaccharides (LPS) injection (endotoxemia model) in kidney and liver, while no significant changes were detected in brain, spleen and lung (Suppl. Fig. S4). However, using cELISA it is not possible to discriminate among the individual members of this family of peptides. Therefore, a specific LC-MS/MS method was developed in order to measure the individual peptides, i.e. pepcan-12 to pepcan-23. Although pepcan-14, -15, -17, -20 and -23 have previously been detected using a very sensitive immunoaffinity MALDI TOF-TOF LC-MS/MS method^27^, LC-MS/MS measurements using optimized protein precipitation and solid phase extraction indicated that only pepcan-23 and pepcan-12 occur at quantifiable and physiologically relevant levels in tissues. The high amounts of pepcan-12 in liver, adrenals, spleen and kidney are noteworthy as they exceed the levels in brain (Fig. 4A). As shown in Fig. 4B, the levels of pepcan-23 were about three times higher than pepcan-12 in the brain. Importantly, the nonapeptide hemopressin was not identified in any of the tissues as acidic conditions during workup were avoided (see discussion). Given the reported protective role of CB2 receptors in the acute immune response and in sepsis^33, 34^, we measured the peptides upon LPS challenge in Swiss albino female mice (eight weeks old). The LC-MS/MS quantification displayed the same trend of pepcan-12 levels as observed with the cELISA quantification (Suppl. Fig. S4), showing a significant increase induced by LPS treatment in kidney, liver and adrenals (Fig. 4A), but no effect in spleen and brain. Overall, pepcan-23 levels were highest in liver and spleen (Fig. 4B). In contrast to pepcan-12, pepcan-23 levels were not affected to the same degree by LPS treatment, despite a minor increase in the liver (Fig. 4B). These data exhibit a relatively uniform pepcan tissue distribution in liver, adrenals, spleen and kidney in the range of 20–130 pmol/g tissue. Interestingly, Pepcan-23 was below the limit of detection in adrenals, which however contained high amounts of pepcan-12. Noteworthy, the spleen expressing high levels of CB2 receptor contained comparably high amounts of pepcan-12 and pepcan-23 as the liver.

### Increase of pepcan-12 upon ischemia reperfusion injury in liver and kidney

**Figure 5:**
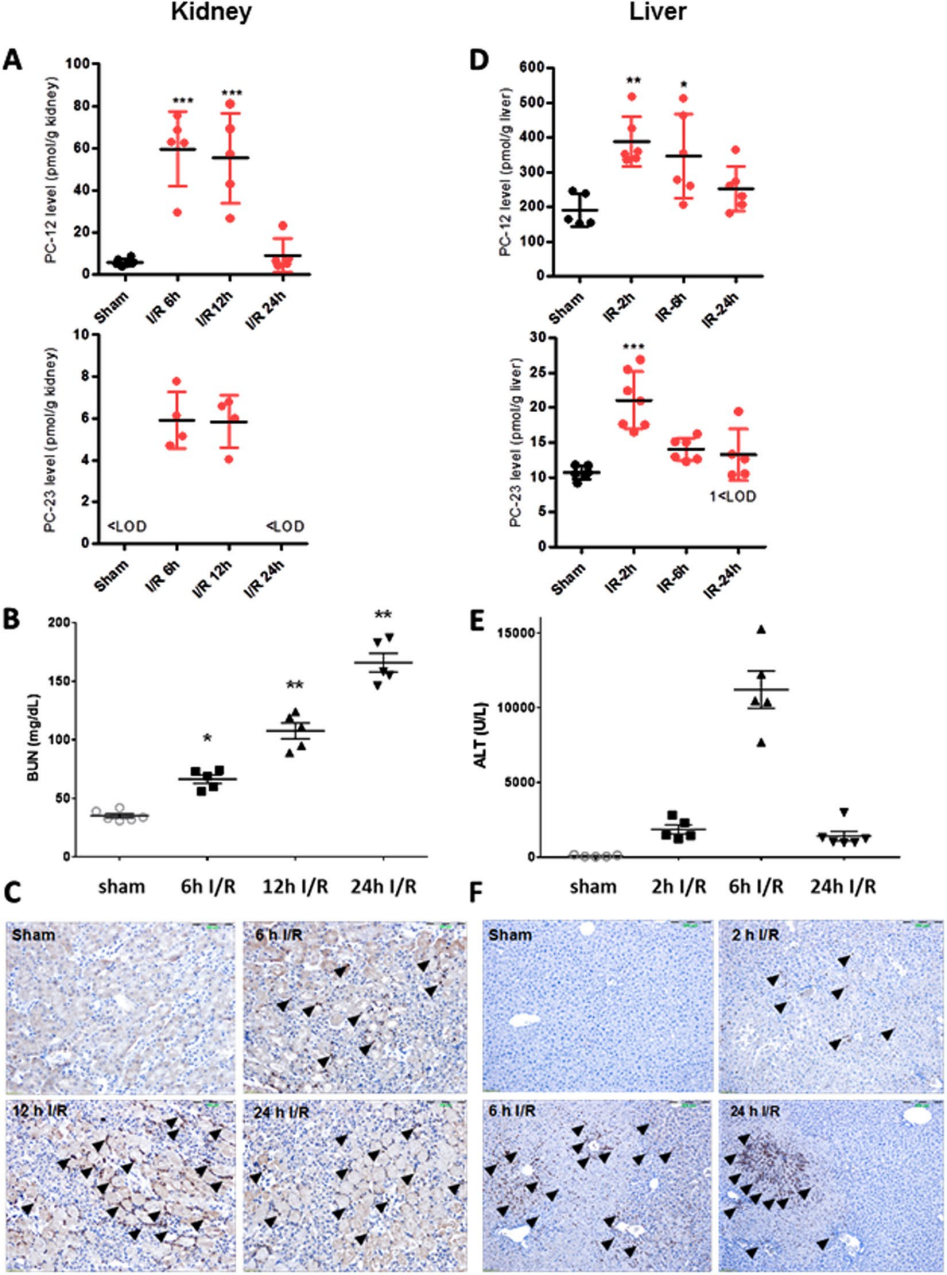
LC-MS/MS measurements of pepcan-12 and pepcan-23 in kidney and liver upon ischemia-reperfusion (I/R) damage and associated pathophysiology. (**A**) Time-dependent increase of PC-12 and PC-23 in kidney upon I/R damage in C57BL6 male mice. Bars show mean values ± SD., ***p < 0.001 vs. sham. < LOD, below limit of detection. (**B**) Time-course of renal I/R injury related blood urea nitrogen (BUN) levels, data show mean values ± SEM, n = 5, one-way ANOVA, *p < 0.05, **p < 0.01 vs. sham operated. (**C**) Representative micrographs of kidney samples after renal I/R injury at different time points after reperfusion (6 h, 12 h, 24 h). Arrowheads show Ly6G positive neutrophil granulocytes infiltrating the kidney following ischemic insult. Scale bar: 200 µm. (**D**) Time-dependent increase of PC-12 and PC-23 in liver upon I/R damage. Bars show mean ± SD. *p < 0.05, **p < 0.01 vs. sham. < LOD, below limit of detection. (**E**) Time-course of hepatic I/R injury related alanine aminotransferase (ALT) levels (units/liter), data show mean values ± SEM, n = 5–6, one-way ANOVA, **p < 0.01 vs. sham operated. (**F**) Representative micrographs of liver samples after hepatic I/R injury at different time points after reperfusion (2 h, 6 h, 24 h). Arrowheads show Ly6G positive neutrophil granulocytes infiltrating the liver following ischemic insult. Scale bar is 200 µm.

In order to investigate whether a change in pepcan levels occurs in established models of tissue injury, pepcans were quantified in mice subjected to sham operation and ischemia/reperfusion (I/R) injury in both liver and kidney (Fig. 5), where CB2 receptors are known to exert protective roles.11,35 As shown in Fig. 5A, renal levels of pepcan-12 increased upon 6 h and 12 h of I/R injury, returning to basal level after 24 h. This concurred with the pathophysiological data (increase of BUN and neutrophil granulocyte infiltration) (Fig. 5B and C). The same was observed upon hepatic I/R damage (Fig. 5D) where the kinetics of pepcan production partially correlated with the pathophysiological parameters (increase and decrease of ALT and neutrophil granulocyte infiltration) (Fig. 5E and F). Because data from cELISA (Suppl. Fig. S4) showed a comparable kinetics of pepcan-12 production (Fig. 5), pepcan-12 seems to be the most relevant pepcan generated upon tissue damage in these tissues. However, in both I/R models the levels of pepcan-23 significantly increased at early times of reperfusion, returning to basal level (liver) or below limit of detection (kidney) as in the sham-operated mice (Fig. 5A and D).

**Figure 6:**
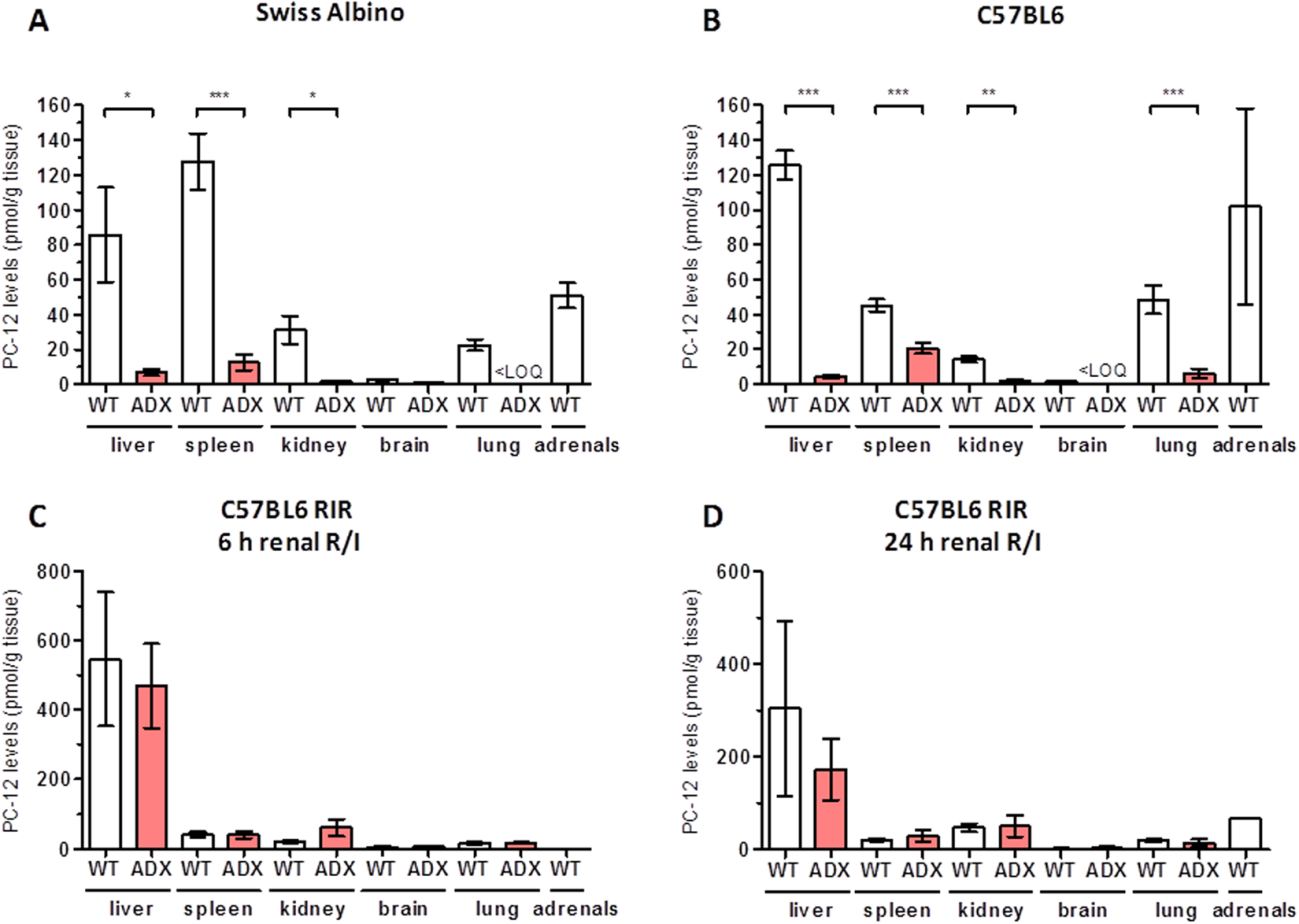
LC-MS/MS quantification of pepcan-12 in different mouse tissues in wild type and adrenalectomized mice. Levels of PC-12 quantified in wild type (WT) and adrenalectomized (ADX) female Swiss albino mice (**A**) and female C57/BL6 mice (**B**). Tissue levels of pepcan-12 quantified in an animal model of renal ischemia reperfusion (renal I/R) after (**C**) 6 h and (**D**) 24 h of reperfusion in ADX C57/BL6 male mice. Data shows means and SD. Groups were compared using two-tailed *t*-Student’s; *p < 0.05; **p < 0.001; ***p < 0.000. < LOQ = below limit of quantification (i.e., detectable but not quantifiable).

Role of adrenals as endocrine gland in the secretion of pepcan-12 under healthy and pathophysiological conditions The exclusive presence of pepcans in cell bodies and axons of a discrete population of noradrenergic neurons in the CNS and in the adrenal medulla was recently shown.28 To address the question whether the adrenals are the site of production of peripheral pepcans, e.g. acting as endocrine gland for pepcan-12, we quantified pepcan-12 in bilaterally adrenalectomized (ADX) mice three weeks post operation. As shown in Fig. 6A and B, in female Swiss albino and female C57BL6 adrenalectomized mice (ADX) the pepcan-12 levels dropped to approximately 10% of the levels measured in control littermates (WT) in liver, kidney and spleen (in C57BL6 only 50%) or disappeared completely in the lung. Remarkably, also levels of pepcan-12 in the brain were significantly reduced upon adrenalectomy, from 2.7 pmol/g to 0.8 pmol/g (Swiss Albino) and 1.8 to below limit of detection (C57BL6) versus WT controls. To further study the modulation of pepcan-12 production under pathological conditions, we compared pepcan levels measured in WT and ADX C57BL6 mice both subjected to renal I/R. Unexpectedly, upon I/R the levels of pepcan-12 in peripheral tissues did not change significantly between WT and ADX mice (Fig. 6C and D). Pepcan-12 concentration in liver was approximately 2–5 times higher upon I/R than in basal condition in both WT and ADX mice. However, compared to ADX mice under normal conditions, the levels of pepcan-12 in the liver of ADX mice after I/R injury were more than 50 times higher, indicating additional production in the liver. Noteworthy, the highest amount of pepcan-12 in renal I/R was measured in liver of C57BL6 R mice after 6 h renal I/R injury (approximately 500 pmol/g tissue) and not in the kidney (Fig. 6C).

## Discussion

Here, we describe a first positive allosteric modulator (PAM) of the cannabinoid CB2 receptor, a ubiquitous GPCR that mediates rather diverse tissue protective and immunomodulatory functions and displays pronounced anti-fibrogenic properties, as previously reviewed^11, 35^. The dodecapeptide RVDPVNFKLLSH (RVD-hemopressin, here referred to as pepcan-12) strongly potentiated the effects of two different CB2 agonists (CP55,940 and 2-AG) by stabilizing agonist binding and exhibiting positive cooperativity, significantly increasing [^35^S]GTPγS biding and signaling through cAMP. Intriguingly, pepcan-12 enhanced the potency of 2-AG mediated-CB2 receptor signaling (Gαi) at the level of cAMP inhibition by a factor eight, which could mediate a strong amplification of downstream effects of the second messenger (Table 2). Thus, it is expected that in tissues pepcan-12 facilitates activation of CB2 receptors by low 2-AG concentrations. In agreement with an allosteric mechanism, in absence of a CB2 receptor agonist pepcan-12 did not affect CB2 receptor signaling. Rather unexpectedly, our data show that the action of pepcan-12 on CB2 receptors is opposite to its action on CB1 receptors^21, 27^ involving binding to yet uncharacterized CB receptor allosteric binding sites. Unlike the CB1 receptor^6, 36^, the structure of the CB2 receptor has not yet been solved. Nonetheless, based on a homology model, a potential allosteric binding pocket adjacent to the CB2 receptor orthosteric ligand-binding site has been postulated^37^. Given that pepcan-12 modulates both CB1 and CB2 receptors, but does not interact with other endocannabinoid receptors such as GPR55^27^, it is feasible that its putative allosteric binding site is easily accessible from the extracellular site and located in an aa sequence (e.g. loop) conserved in both CB receptors.

Pepcan-12 (RVD-hemopressin) was originally reported to act as CB1 receptor agonist because it increased intracellular Ca2 + levels in HEK-293 cells co-expressing chimeric G16/Gi3 and CB1 receptors, mediated phosphorylation of ERK1/2, and induced CB1 internalization^26^. It was shown that the peptide bound to CB1 receptors in cerebellar membranes and could displace the binding of the [^3^H]CP55,940 with nanomolar affinity, although to a lesser extent than the classic CB1 receptor ligand SR141716. However, judging the binding data in this paper, RVD-hemopressin displayed only partial displacement (approximately 55%) of the orthostertic ligand [^3^H]CP55,940^26^. Subsequently, in a more detailed study we have shown that pepcan-12 (RVD-hemopressin), which was identified together with a whole family of N-terminally extended peptides, acts as a CB1 receptor NAM and does not act like an agonist at the level of major signaling pathways (cAMP) *in vitro* ^27, 38^. Our findings were independently confirmed in a physiologically more meaningful setup with cultured hippocampal neurons measuring neurotransmission by electrophysiology^21^. Similar to the synthetic CB1 receptor NAMs ORG2756921 and PSNCBAM-122, also pepcan-12 attenuated 2-AG mediated DSE but did not directly inhibit CB1 receptors^21^. While the action of pepcan-12 as CB1 receptor NAM was clearly observed neither pregnenolone nor hemopressin did modulate DSE.

Unfortunately, studies with hemopressin-like peptides (i.e. pepcans) have faced different challenges. For instance, the handling of these peptides is hampered by aggregation^27, 38^. Moreover, the metabolic stability of these peptides is limited *in vivo* and data from uncontrolled pharmacological experiments performed in mice^39^ are difficult to interpret without pharmacokinetics, which may have added to the confusion in the literature^40^. Although different *in vivo* studies have been carried out with hemopressin and RV-hemopressin^39, 40^, the only endogenous peptides present at physiological levels appear to be pepcan-12 (i.e., RVD-hemopressin) and pepcan-23 as reconfirmed by the present study. The latter is postulated to be a precursor of pepcan-12 (i.e. pro-peptide) as it is not directly modulating CB receptors but is found abundantly in brain, liver, spleen and kidney. Although hemopressin was never detected as endogenous molecule in tissues by immunoaffinity LC-MS/MS^27^ or LC-MS/MS in this study, it is readily generated in an acidic environment from pepcan-12 or even longer pepcans (i.e. pepcan-23) during experimental workup due its acid sensitive cleavage site between aspartic acid and proline^27, 41^. Thus, it is unfortunate that hemopressin is specified^38^ or sold as “endogenous peptide” by commercial providers because its presence as physiological peptide has so far not been established. It is possible that administered hemopressin exerts effects different from pepcan-12 or, even more likely, that it competes as substrate with degrading enzymes for pepcan-12 *in vivo*, thus potentially increasing endogenous pepcan-12 levels.

In this study, we provide first evidence on the presence of physiologically relevant amounts of pepcan-12 and pepcan-23 in different peripheral mouse tissues. It is noteworthy that although pepcan-23 is present in brain it is absent in adrenals, which might be related to distinct enzymatic processing or half-lives of these peptides. The adrenal medulla, which is a major paraganglion of the sympathetic nerve and executes the systemic release of adrenalin and trace noradrenalin upon stress was previously shown to contain significant amounts of pepcan-12^28^. Because the adrenals are involved in the secretion of pepcan-12, as shown here, it clearly exhibits characteristics of a peptide hormone. Indeed, under normal conditions, the majority of pepcan-12 detected in peripheral tissues appears to have its origin in the adrenals. The tissue levels of pepcan-12 in ADX mice fed with normal diet and 0.9% NaCl drinking water diminish by approximately 90% (Fig. 6). Unexpectedly, pepcan-12 was also significantly reduced in the brain, suggesting a possible connection between these organs and an important role of the adrenals in the generation of basal pepcan-12 in brain.

To better understand the pathophysiological relevance of pepcans we studied their regulation in two independent disease models. CB2 receptors are strongly implicated in acute and chronic immune modulation^33, 34^ and we assessed the effects of endotoxemia in Swiss albino mice on the levels of pepcan-12 and pepcan-23. Upon LPS challenge, pepcan-12 was significantly increased in liver, kidney and adrenals (Fig. 4). The effect was most obvious in kidney. No effect was seen in brain or spleen (Fig. 4A). Conversely, pepcan-23 levels were not modulated, despite a minor increase in the liver (Fig. 4B). It is feasible that the enzymatic generation of pepcans from alpha hemoglobin is related to multiple enzymes with distinct kinetics and this is reflected in the above analyses. With the present LC-MS/MS method we obtained lower absolute amounts of pepcan-12 in brain and adrenals than in our previous study^28^, which can be explained by the difference in the extractions procedure using solid phase extraction clean-up to minimize matrix effects.

The strong increase of this peptide in the liver upon I/R tissue injury cannot be explained by adrenal secretion because in the adrenalectomized littermates the overall amounts did not change significantly. Thus, I/R injury seems to induce the production of pepcan-12 also in the liver where a strong increase was observed (Fig. 6), but we cannot exclude a production of pepcans in kidney. Interestingly, hepatocytes express HBA1/HBA2 (hemoglobin subunit alpha 1/2) in the context of oxidative stress and liver damage^42^.

Pepcans contain an aa sequence exclusively generated from alpha hemoglobin gene expression (with no homology to any other gene). So far, we did not obtain any experimental evidence supporting the hypothesis that basal pepcan production is related to hemoglobin degradation (i.e. hemolysis). Thus, our data challenge the notion that these peptides might have a proteasomal orgin^41^, although hemolysis and proteasomal peptide cleavage cannot be excluded during tissue damage, e.g. in the liver. As indicated by immunohistochemistry, in brain and adrenals pepcans appear to be expressed in noradrenergic cells independently of the presence of alpha hemoglobin protein^28^, possibly through the *de novo* expression and fast differential processing of the HBA1/HBA2 gene products.

Here we provide a first comprehensive distribution of pepcan-12 in peripheral tissues, with adrenals, liver and spleen showing the highest amounts (50–130 pmol/g tissue), followed by kidney and lung (20–60 pmol/g) and brain (2–3 pmol/g tissue). The analytical data indicate that pepcan-23 is present at significant amounts (10–15 pmol/g) in the brain (Fig. 4B), exceeding the amounts of pepcan-12. Thus, the data from cELISA published previously^27^ likely reflect the absolute amounts of both pepcan-12 and pepcan-23. Moreover, this finding strongly suggests that pepcan-23 could be the pro-peptide of pepcan-12 and might be the pepcan species stained in the abundant locus coeruleus projecting fibers in the brain^28^.

**Figure 7:**
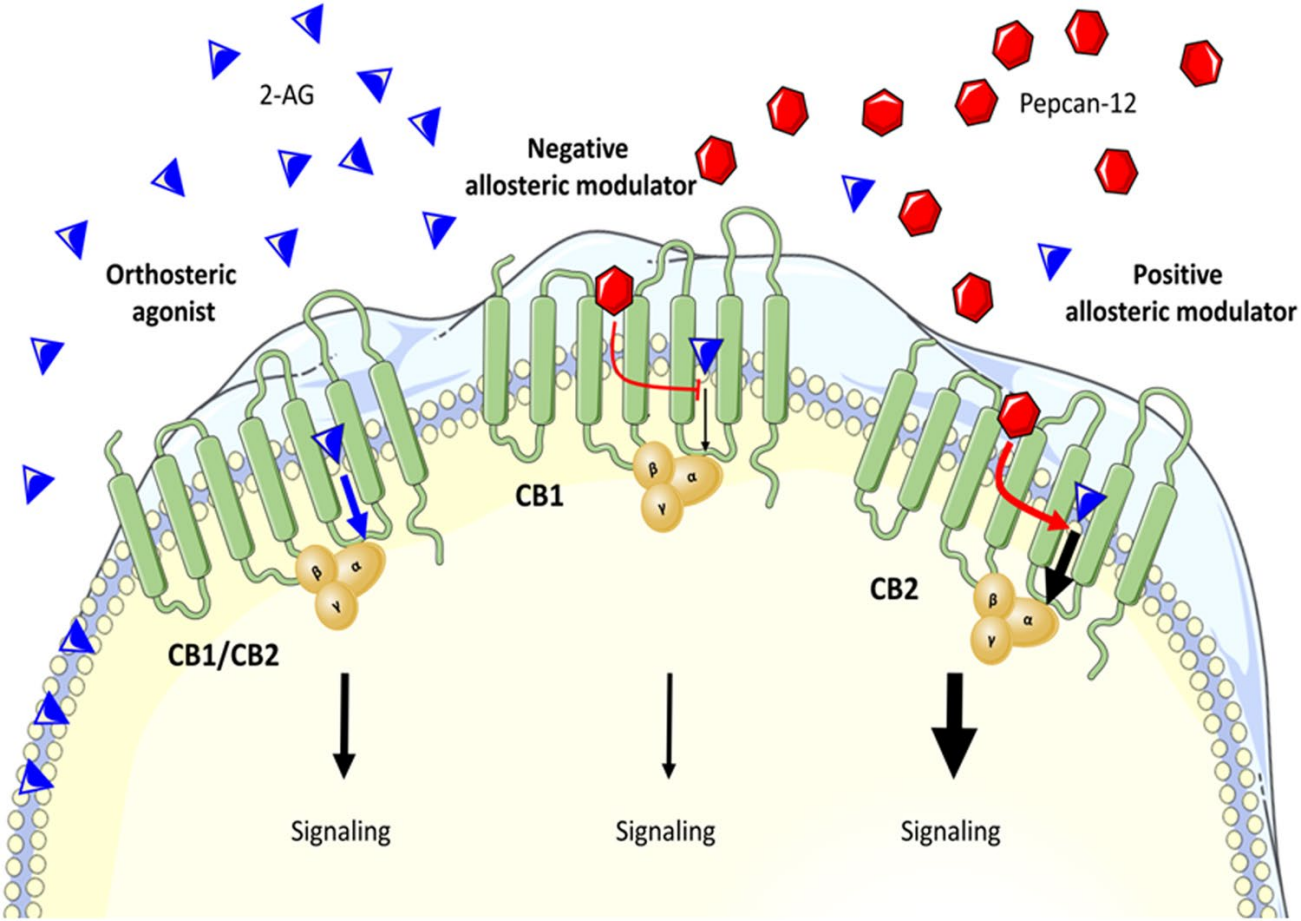
Illustration showing the opposite effects of pepcan-12 on CB1 and CB2 receptor G protein signaling. In presence of the endocannabinoid 2-AG, pepcan-12 acts as a negative allosteric modulator (NAM) of CB1 and as a positive allosteric modulator (PAM) of CB2. This illustration was drawn using Microsoft Power Point 2010.

Upon tissue damage pepcan-12 was increased time-dependently as found in experiments involving I/R damage (Fig. 5). The increase of the endocannabinoids AEA and 2-AG in the same I/R mouse models has been shown previously^43^, suggesting that pepcan-2 could potentiate CB2 receptor signaling through endocannabinoids as protective mechanism. The beneficial effects of CB2 receptor activation in different pre-clinical models of inflammation and pain is well documented in the literature^11, 13, 14, 33–35, 43^. But also inverse agonists show positive effects in certain cellular systems and animal models^44^. Moreover, some ligands can behave either as agonists or as inverse agonists depending on the proportion of constitutively active receptors (defined as “protean agonists”)^45^. An additional level of complexity in GPCR activation include the concept of functional selective ligands (or biased agonists)^32^. The existence of endogenous allosteric peptide modulators of CB receptors could thus implicate an additional important level of tissue and disease-dependent endogenous control over the ECS. In light of the overall beneficial effects of CB2 receptor agonists and in parts detrimental effects of CB1 receptor agonists in peripheral pathophysiological processes^15–17, 35, 43^, the physiological relevance of pepcan-12 should be addressed in more detail in future experiments. Thus, the role of pepcan-12 could be related to shifting the CB1/CB2 receptor activation ratio towards CB2 receptor activation (Fig. 7), which would have beneficial effects in kidney and liver, possibly also other peripheral organs. As recently reviewed for CB1 receptor NAMs^46^ our study now uncovers the first allosteric mechanism for the CB2 receptor. Despite the wide range of possibilities for CB2 receptor modulation known, to our knowledge, pepcan-12 represents the first allosteric modulator for this receptor.

## Materials and Methods

### Materials

Analytical standards PVNFKLLSH (Hemopressin, pepcan-9), RVDPVNFKLLSH (Pepcan-12, PC-12, RVD-Hemopressin), HKLRVDPVNFKLLSH (Pepcan-15, PC-15), HAHKLRVDPVNFKLLSH (Pepcan-17, PC-17), SDLHAHKLRVDPVNFKLLSH (Pepcan-20, PC-20) and SALSDLHAHKLRVDPVNFKLLSH (Pepcan-23, PC-23) were synthesized by Genecust Europe. The internal standard RVDPVNFKFL (5,5,5-D3) SH (PC-12-d_3_) was synthesized by SHBC. The proteases inhibitor cocktail was obtained from Roche applied science (Complete Ultra Tablets Mini). HPLC-grade methanol, acetonitrile and formic acid were obtained from Sigma-Aldrich, Steinheim, Germany. Deionized water (18.2 MΩ x cm) was obtained from an ELGA Purelab Ultra Genetic system (VWS (UK) Ltd, ELGA LabWater, UK).

### [^35^S]GTPγS assay

Assays were performed as previously described^47^. Clean membranes expressing *h*CB2 were diluted in silanized plastic tubes with GTPγS binding buffer (50 mM Tris-HCl, 3 mM MgCl_2_, 0.2 mM EGTA and 100 mM NaCl at pH 7.4 supplemented with 0.5% fatty acid-free BSA) in the presence of 10 μM of GDP and 0.1 nM of [^35^S]GTPγS. The mixture was kept on ice until the binding reaction was started by adding the vehicle or compounds. Non-specific binding was measured in presence of 10 μM GTPγS. The tubes were incubated at 30 °C for 90 min under shaking and then put on ice to stop the reaction. An aliquot (185 μL) of the reaction mixture was rapidly filtered through a 96-well microplate bonded with GF/B glass fibre filters previously pre-soaked with ice-cold washing buffer (50 mM of Tris-HCl pH 7.4 plus 0.1% fatty acid-free BSA). The filters were washed six times with 180 µL of washing buffer under vacuum. The radioactivity was measured after the addition of 45 µL of MicroScint20 scintillation cocktail. Specific binding was calculated by subtracting the residual radioactivity signal obtained in presence of an excess of GTPγS and the results were expressed as % of vehicle control.

### cAMP assay

cAMP assays were performed in CHO-K1-*h*CB2 stably transfected with the pGloSensorTM 22-F plasmid (Promega) as previously described^48^. Briefly, cells were seeded in 96-well plate and after attachment the medium was replaced by 40 µL of equilibration medium (GloSensor™ cAMP Reagent diluted in 5% v v^−1^ of CO_2_-independent medium plus 10% FBS). The plates were incubated for 2 h at RT in the dark. Compounds or vehicle were diluted in 10 µL assay medium containing 1 µM of forskolin and 250 µM of 3-isobutyl-1-methylxanthine (IBMX). Chemiluminescence was monitored continuously starting from immediately after the addition and for 30 min (keeping the plate at RT and in the dark). Basal levels of chemiluminescence (cells plus IBMX and vehicle without forskolin) were subtracted to the measured values. Results were normalized by subtracting the residual cAMP production and expressed as % of vehicle control.

### β-arrestin assay

The PathHunter^®^ β-arrestin cell line (CHO-K1-HOMSA-CNR2) was maintained in culture in F-12 medium supplemented with 10% FBS, 200 μg/mL hygromycin and 800 μg mL^−1^ Geneticin (G418). For the experiments, 100 μL well^−1^ of cell suspension (2 × 10^5^ cell/mL) was plated in 96-well plate and left overnight in the incubator. The day after, tested compounds or vehicle were diluted in 40 μL of CO_2_-independent medium plus 10% FBS, added to the cells and incubated for 90 min at 37 °C. Following stimulation, signal was detected using the PathHunter® detection Kit according to the instruction protocol and the chemiluminescence was measured. Background levels were subtracted and results expressed as % of vehicle control.

### Competitive ELISA (cELISA)

Maxisorp microtiter 96 wells plates (Nunc) were coated by incubation with 100 μL of 1 µg/mL synthetic pepcan-12 (Genscript) in PBS pH 7.2 at 4 °C O/N. Washing of peptide excess and blocking of non-specific sites was performed with PBS-T 0.05% pH 7.2 and PBS-T 0.05% + 1% BSA fatty acid free respectively. Calibration concentration points (0.01-0.1-1-3-10-30-100-1000 nM) or sample were pre-incubated with 0.0125 nM of 1A12 mAb for 30 min at room temperature and added to the wells and incubated for 2 h at room temperature. After washing, plates were incubated at room temperature for 60 min with 50 μL HRP- conjugated secondary antibody goat anti-mouse IgG (Bio-Rad). Plates were finally developed with 50 μL of 3,3′,5,5′-tetramethylbenzidine substrate (Pierce) after washing. This reaction was stopped with 50 μL of 2 M H_2_SO_4_ after sufficient colour development (usually after 20 min). The OD of the reaction product was then recorded at 450 nm. Sample preparation: mouse tissues were weighted, homogenized in PBS + Protease inhibitor cocktail (Roche) and two times their volume of acetonitrile were added. After centrifugation at maximum speed for ten min, the supernatant was collected, dried in a speed vacuum and resuspended in 200 μL of PBS.

### Radioligand Binding Assays

Radioligand binding assays using [^3^H]CP55,940 and [^3^H]WIN55,212-2 were performed with CB2 receptor membrane preparations as reported previously^27^. Briefly, 20 μg of membranes of CHO-K1-*h*CB2 cells were incubated in 500 μL of binding buffer (50 mM Tris-HCl, 2.5 mM EDTA, 5 mM MgCl_2_, 0.5 mg/mL fatty acid-free BSA, pH 7.4) in silanized glass tubes in presence of different concentrations of pepcans and 0.5 nM [^3^H]CP55,940 (168 Ci/mmol) or 2.5 nM [^3^H]WIN55,212-2 (40 Ci/mmol) for 60 min at 30 °C. Nonspecific binding of the radioligand was determined in the presence of 10 μM WIN55,512-2 or CP55,940, respectively. After the incubation time, membrane suspensions were rapidly filtered through a 0.1% polyethyleneimine presoaked 96-well microplate bonded with GF/B glass fiber filters (UniFilter-96 GF/B, PerkinElmer Life Sciences) under vacuum and washed 12 times with 150 μL of ice-cold binding buffer Filters were added with 40 μL of MicroScint20 scintillation liquid, and radioactivity was measured with the Trilux top counter (PerkinElmer Life Sciences). Data were collected from 3–10 independent experiments, each performed in triplicate, and the nonspecific binding was subtracted. The results are expressed as a percentage of vehicle-treated samples.

### Dissociation Kinetic Studies Using Isotopic Dilution

Dissociation kinetics experiments were performed as described before^27^. Briefly, 20 μg of membranes of CHO-K1-*h*CB2 cells were incubated in 500 μL of binding buffer in silanized glass tubes and incubated with 0.5 nM [^3^H]CP55,940 for 60 min at 30 °C. Radioligand dissociation was initiated by the addition of 1 μM WIN55,212-2 in the presence of 300 nM pepcan-12 or solvent (DMSO) and incubated for 1–120 min, at 30 °C. Nonspecific binding of the radioligand was determined in the presence of 10 μM CP55,940. After the incubation time, membrane suspensions were rapidly filtered through a 0.1% polyethyleneimine presoaked 96-well microplate bonded with GF/B glass fiber filters and washed as described above.

### Equilibrium Binding Studies Using a Fluorescently Labeled Pepcan-12 Derivative

The equilibrium dissociation constant (Kd value) of the fluorescein-labeled pepcan-12 derivative pepcan-F4 was determined using CHO-K1-*h*CB2 cells as previously described^27^. Equilibrium saturation binding was determined experimentally, and maximal specific binding (saturation) was reached already at 5 min at 37 °C. Different concentrations of pepcans and 100 nM pepcan-F4 were incubated together with 105 cells suspended in 200 μL of binding buffer in silanized plastic tubes, under shaking for 60 min at 37 °C. After centrifugation at 4 °C and washing with ice-cold binding buffer, the cells were resuspended in ice-cold PBS and transferred into 96-well black plates, where the fluorescence intensities were measured using a Tecan Farcyte reader (485 nm excitation/535 nm emission). The specific binding was determined after washing and subtracting the nonspecific fluorescence calculated in parallel experiments carried out in non-transfected CHO cells.

### Animals

The investigation conforms to the Guide for the care and use of laboratory animals published by the US National Institutes of Health (NIH, publication No. 85–23, revised 1996) and was approved by the Institutional Animal Care and Use Committee of the National Institute on Alcohol Abuse and Alcoholism (NIAAA). Animal experiments complied with Swiss regulations and were carried out in accordance with relevant Swiss guidelines and regulations and approved by the Kantonale Amt für Landwirtschaft und Natur, Veterinärdienst. Male C57Bl6/J mice (weighting between 25–30 g) and female Swiss albino mice (8 weeks) were purchased from The Jackson Laboratory (Bar Harbor, ME, USA and Janvier Labs) and were housed in a room maintained at 12 h light-dark cycles and at constant temperature and humidity. Mice had free access to standard chow and drinking water. Adrenalectomized (ADX) mice were purchased from The Jackson Laboratory and male C57BL6/J mice were subjected to bilateral adrenalectomy by the vendor. When surgery was completed, mice were held by the vendor for approximately one week to allow wound healing before they delivered. ADX mice showed neither signs of distress nor unusual behavior. The only diet adjustment that had to be arranged was to provide 0.9% NaCl in drinking water ad libitum instead of normal tap water.

### Mouse model of endotoxemia

Swiss albino female mice were housed in groups of five per cage in a selected pathogen-free unit under controlled 12-h light/12-h dark cycle, ambient temperature 21 °C ± 1 °C and 40% to 50% humidity with free access to standard rodent chow and water. The mice were acclimatized for at least 3 days before the experiment. Swiss albino mice (females, 8 weeks old) were injected intraperitoneally (i.p.) with LPS (5 mg/kg) and sacrificed after 2 h. LPS was serotype 055:B5 (Sigma Aldrich, Switzerland).

### Mouse model of renal ischemia reperfusion injury

Mice underwent unilateral kidney ischemia reperfusion as described elsewhere^49^ (Hamar P., Proc Natl Acad Sci USA. 2004). Briefly, anesthesia was induced by the intraperitoneal administration of ketamine (100 mg/kg) and xylazine (10 mg/kg). After midline laparotomy, right kidney was removed in all cases to prevent compensation. The left renal pedicle was isolated and clamped for 25 min followed by reperfusion for 6 h, 12 h or 24 h according to the experimental setting. After the intervention, abdominal cavity was closed by using 6–0 vicryl suture (Ethicon, Somerville, NJ, USA) and mice were transferred into clean cages and allowed to recover. As post-operative care, buprenorphine was administered subcutaneously (Buprenorphine SR ZooPharm, Wildlife Pharmaceuticals, Fort Collins, CO, USA). Sham operated animals underwent the above-mentioned procedures except of left kidney clamping. At the end of reperfusion, mice were sacrificed by cervical dislocation, Blood was collected via cardiac puncture, tissues and post-ischemic kidneys were snap frozen in liquid nitrogen and kept at −80 °C until further use. Tissue samples were collected and fixed in 10% buffered formalin for histopathological evaluation as well. The function of the injured left kidney was assessed by measuring blood urea nitrogen (BUN) level in serum samples (IDEXX VetTest® Chemistry Analyzer and BUN test strips; Westbrook, ME, USA).

### Mouse model of hepatic ischemia reperfusion injury

Partial hepatic ischemia reperfusion was performed as described by Batkai *et al*.^13^. Briefly, following anesthesia, the liver was exposed by midline laparotomy. The vascular pedicle common to the median and left-lateral lobes was clamped. In this model of partial hepatic ischemia, mesenteric venous congestion was prevented by allowing portal decompression throughout the right and caudate lobes of liver. The duration of hepatic ischemia was 1 h followed by 2 h, 6 h or 24 h reperfusion as indicated. Sham surgeries were completely identical except of hepatic vascular pedicle clamping. At the end of reperfusion, blood was collected and liver samples were removed and snap-frozen in liquid nitrogen as described above. Serum aspartate amino-transferase (AST) and alanine amino-transferase (ALT) levels were determined in serum samples (IDEXX VetTest® Chemistry Analyzer and AST and ALT test strips) in order to assess hepatic injury.

### Histological examination of tissue sections

Tissue samples were fixed in 10% buffered formalin. After embedding and sectioning 5 µm slices, samples were stained with the conventional haematoxylin/eosin staining method.

### LC-MS/MS for pepcans

#### Sample preparation

Tissue (~50 mg) were homogenized on a BeadBeater (Mini-BeadBeater-24, BioSpec, Oklahoma, USA) using 2 mL Microcentrifuge vials (XXTuff Microvials, BioSpec, Oklahoma, USA) and Chrome-Steel Beats (2.3 mm dia, BioSpec, Oklahoma, USA) with 200 µL of Tris buffer + 1 mM EDTA + protease Inhibitor cocktail (prepared according to manufacturer’s instructions-Roche) and 10 µL internal standard solution of 1 µg/mL (IS). After homogenization 200 µL of acetonitrile was added and samples were kept on ice for 30 min to facilitate protein precipitation. After centrifugation at 16,100 × g at 4 °C for 20 min the supernatant was transferred to a 2 mL silanized tubes containing 1.6 mL of distilled water (10% final concentration of acetonitrile) and cleaned up through Oasis® HLB 1cc (30 mg) cartridges previously conditioned (1 ml methanol, followed by equilibration with 1 mL 10% ACN). The sample was loaded and subsequently washed with 1 mL 10% ACN. Peptides were eluted with 70% ACN. The eluates were evaporated to dryness in a SpeedVac and extracts reconstituted in 50 µL of 10% ACN.

#### Chromatographic and mass spectrometric conditions

Analyses were conducted on a LC-MS/MS system consisting of an API 4000 QTrap mass spectrometer equipped with a TurboIonSpray probe (AB Sciex Concord, Ontario, Canada) connected to a Shimadzu UFLC (Shimadzu Corporation, Kyoto, Japan). Data acquisition and analysis were performed using Analyst software version 1.6 (AB Sciex Concord, Ontario, Canada). The TurboIon Spray interface was operated in positive mode. The parameters of the source using nitrogen as curtain gas were the following: +2000 V, temperature 650 °C, curtain gas 25 psi, GS1 50 psi and GS2 50 psi. The entrance potential and collision cell exit potential were set to 10 V. Analytes were measured in multiple reaction-monitoring mode (MRM). The following MRM transitions were considered for the quantification of pecan-12 and pepcan-23: PC-23 520.4 → 611.6, 433.8 → 489.6, PC-12 475.6 → 110.4, 356.9 → 430.3, 356.9 → 256.5. Chromatographic separation of the peptides (molecular weight range from 1423.4 to 2597 Da) was performed on a C8-2 3 µm ReproSil-Pur Basic® (Maisch). Mobile phase A was water containing 0.1% formic acid (by volume) and mobile phase B was acetonitrile containing 0.1% formic acid. Peptides were eluted using a linear gradient from 2% to 80% organic solvent over 19 min at 0.3 mL/min and an oven temperature of 40 °C. The results were analysed based on the peak area ratio between the analyte and IS. Values obtained for R^2^ (GraphPad Prism 6, linear regression) were higher than 0.97 and the recoveries of the analytes were between 70–100%.

### Data Availability

All data generated or analysed during this study are included in this published article (and its Supplementary Information files).

## Electronic supplementary material

Supplementary Figures
