# Supplementary material for: Pepcan-12 (RVD-hemopressin) is a CB2 receptor positive allosteric modulator constitutively secreted by adrenals and in liver upon tissue damage
Source: Sci Rep. 2017 Aug 25;7:9560. doi: 10.1038/s41598-017-09808-8 (PMC5573408; doi:10.1038/s41598-017-09808-8)
Supplement: Supplementary file 1 — Supplementary Figures [file 41598_2017_9808_MOESM1_ESM.pdf]

Pepcan-12 (RVD-hemopressin) is a CB2 receptor positive allosteric modulator constitutively secreted by adrenals and in liver upon tissue damage

Vanessa Petrucci[1#], Andrea Chicca[1#], Sandra Glasmacher[1], Janos Paloczi[2], Zongxian Cao[2], Pal Pacher[2], Jürg Gertsch[1*]

[1]Institute of Biochemistry and Molecular Medicine, University of Bern, Bühlstrasse 28, 3012 Bern, Switzerland

[2]Laboratory of Cardiovascular Physiology and Tissue Injury, National Institutes of Health/NIAAA, Bethesda, MD, USA

Supplementary Figure S1.
Effect of pepcans on CB2 receptor signaling in absence of agonist.

Supplementary Figure S2.
Effect of N-terminally extended pepcans on CB2 receptor-mediated cAMP production.

Supplementary Figure S3.
Effect of pepcan-12 on CB2 receptor-mediated beta-arrestin recruitment by CP55.940.

Supplementary Figure S4.
cELISA quantification of pepcans in LPS-stimulated versus normal (saline) Swiss albino female mice.

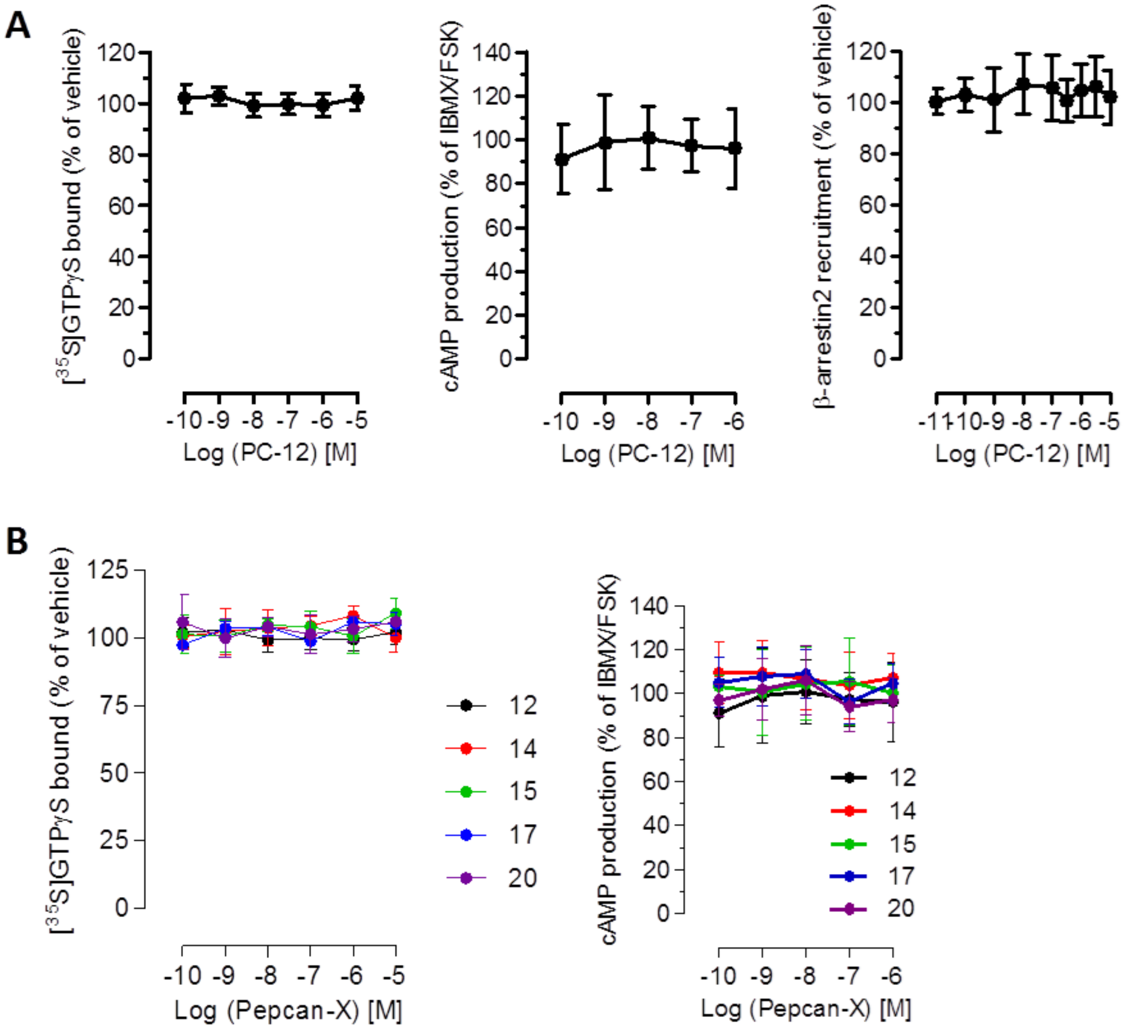

Figure S1. Effect of pepcans on CB2 receptor signaling in absence of agonist. (A) No effect was observed by pepcan-12 on [^{35}S]GTPγS binding, cAMP levels or β-arrestin2 recruitment. (B) Lack of effects of pepcans -14,-15,-17 and -20 on CB2 receptor-mediated [^{35}S]GTPγS binding and cAMP levels. Data show mean values ± SD of at least 3 independent experiments each performed in triplicates.

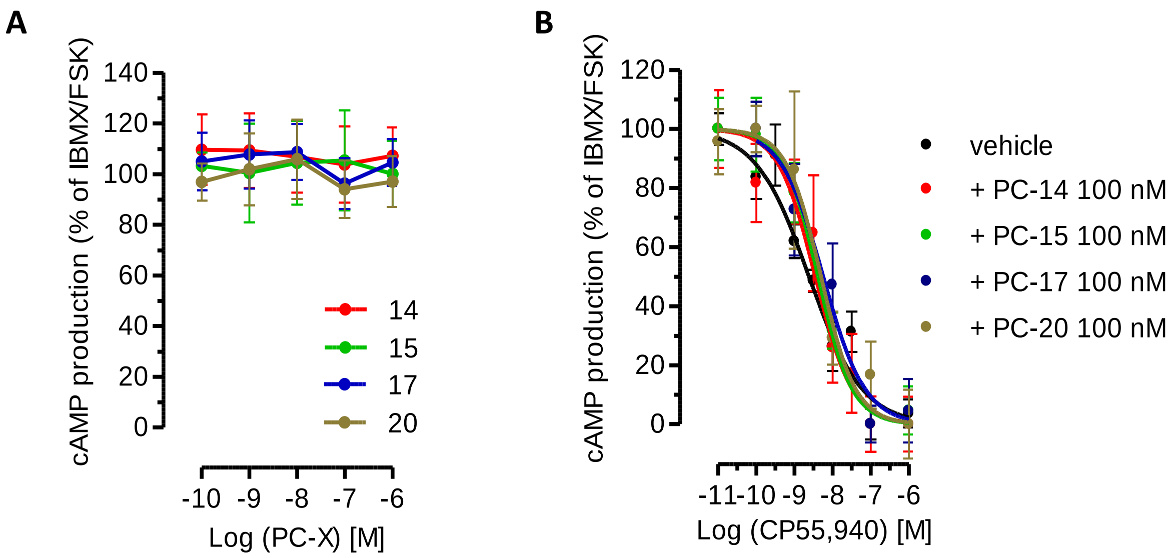

Figure S2. Effect of N-terminally extended pepcans on CB2 receptor-mediated cAMP production. (A) Effect of pepcans -14,-15,-17 and -20 on stimulated cAMP production in absence of a CB2 agonist. (B) Effects of pepcans -14,-15,-17 and -20 on CB2 receptor-mediated binding cAMP levels in presence of various concentrations of CP55,940. Data show mean values ± SD of at least 3 independent experiments each performed in triplicates.

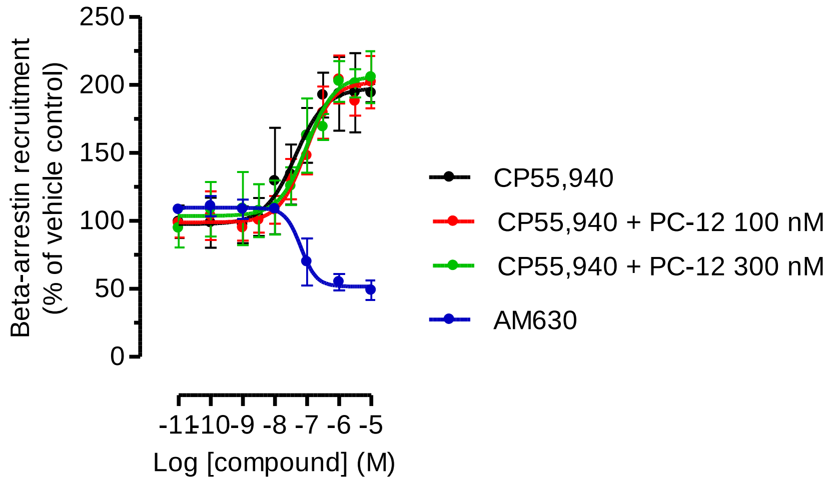

Figure S3. Effect of pepcan-12 on CB2 receptor-mediated beta-arrestin recruitment by CP55.940. Data show mean values ± SD of at least 3 independent experiments each performed in triplicates.

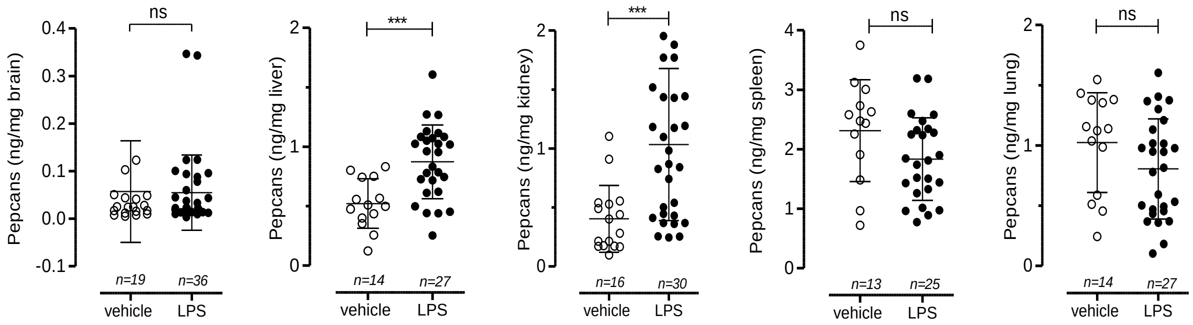

Figure S4. cELISA quantification of pepcans in LPS-stimulated versus normal (saline) Swiss albino female mice. Swiss albino mice (females, 8 weeks old) were injected intraperitoneally (i.p.) with LPS (5 mg/kg) and sacrificed after 2 h. Groups were compared using two-tailed t-Student's; $p<0.0001$; ns= not significant.
